# The glyoxalase system as an example of a cellular maintenance pathway with relevance to aging

**DOI:** 10.18632/aging.100268

**Published:** 2011-01-24

**Authors:** Axel Kowald

**Affiliations:** Humboldt University Berlin, Institute for Biology, Theoretical Biophysics, Invalidenstrasse 42, 10115 Berlin, Germany

In a recent issue of AGING, Scheckhuber et al. studied the effect of methylglyoxal (MG) metabolism on growth and lifespan in *P. anserina* [1]. Methylglyoxal (2-oxopropanal) is generated enzymatically by three types of enzymes (methylglyoxal synthase, cytochrome P450 isozyme and amine oxidase) and non-enzymatically as a side product of glycolysis from dihydroxyacetone phosphate and glyceraldehyde-3-phosphat. MG reacts by Maillard reaction with lysine and arginine residues of proteins and thus leads to advanced glycosylation end products (AGE) [2]. It is removed enzymatically via the glyoxalase system, which consists of two enzymes [3].

On a glucose rich media, overexpression of only one of the two enzymes, *PaGlo1,* led to a reduction of lifespan, while overexpression of *PaGlo1* together with *PaGlo2*, increased lifespan. A situation reminiscent of the superoxide dismutase (SOD), catalase system that is involved in the detoxification of the superoxide radical. There, SOD converts the superoxide radical into hydrogen peroxide, which is then removed by catalase or glutathione peroxidase. Also in that case it has been observed that overexpressing only the first component (SOD) is detrimental instead of increasing resistance to oxidative stress [4,5,6].

In both cases an apparently simply reaction system displays phenotypes that are surprising and can be better understood when modelled mathematically in a systems biological context. Such an approach showed that an alternative reaction pathway is required for the superoxide radical to explain the experimental findings [7] and thus provided a deeper understanding of the possible mechanism. Similarly, also the glyoxalase system might benefit from a modelling approach to test if the hypothesis that the negative effects of oxerexpressing *PaGlo1* alone are mediated via a reduction of free GSH, is plausible. It has also be shown that the mutagenic action of methylglyoxal involves the generation of free oxygen radicals [8]. Since GSH is also involved in the detoxification of reactive oxygen species, its decline would be detrimental for MG degradation as well as the removal of ROS that are generated by MG.

Numerical values are of critical importance for the construction of kinetic models and the absence of such values is often a serious obstacle. However, in erythrocytes the steady state concentrations and kinetic properties of the glyoxalase system have been determined [9,10], which opens the way for a systems biological approach. The work of Scheckhuber [1] thus provides interesting results for further investigations of a cellular maintenance pathway with relevance to aging.
